# Economic impact of enhanced recovery after surgery protocol in minimally invasive cardiac surgery

**DOI:** 10.1186/s12913-021-06218-5

**Published:** 2021-03-20

**Authors:** Johannes Petersen, Benjamin Kloth, Johanna Konertz, Jens Kubitz, Leonie Schulte-Uentrop, Gesche Ketels, Hermann Reichenspurner, Evaldas Girdauskas

**Affiliations:** 1 Department of Cardiovascular Surgery, University Heart & Vascular Center Hamburg, Hamburg, Germany; 2 Department of Anesthesiology, University Hospital Hamburg-Eppendorf, Hamburg, Germany; 3 Physiotherapy, University Hospital Hamburg-Eppendorf, Hamburg, Germany

**Keywords:** Enhanced recovery after surgery, ERAS, Perioperative care, Minimally invasive cardiac surgery, Cost analysiss

## Abstract

**Background:**

ERAS (Enhanced Recovery After Surgery) is a multidisciplinary and integrative approach with the goal of optimizing the postoperative recovery. We aimed to analyze the economic impact of a newly established ERAS protocol in minimally invasive heart valve surgery at our institution.

**Methods:**

ERAS protocol was implemented in 61 consecutive patients who were referred for elective minimally-invasive aortic or mitral valve surgery, between February 1, 2018 and March 31, 2019 (**ERAS-group**). Another 69 patients who underwent elective minimally-invasive heart valve surgery during the same time period were managed according to the hospital standards (**Control-group**). A detailed cost comparison analysis was carried out from a hospital perspective using a micro-costing approach.

**Results:**

The total in-hospital stay was significantly shorter in the ERAS-group compared to the Control-group (6.1 ± 2.6 vs 7.7 ± 3.8 days; *p* = 0.008) resulting in significant cost savings of €1087.2 per patient (*p* = 0.003). Due to the intensified physiotherapy in the ERAS protocol, the costs for physiotherapy were €94.3 higher compared to the Control-group (*p* < 0.001). The total costs in the ERAS cohort were €11,200.0 ± 3029.6/patient compared to € 13,109.8 ± 4527.5/patient in the Control-Group resulting in cost savings of €1909.8 patient due to the implementation of the ERAS protocol (*p* = 0.006).

**Conclusion:**

Implementation of an ERAS-protocol in minimally-invasive cardiac surgery can be carried out safely with a fast postoperative recovery of the patient. ERAS results in a financial benefit of up to €1909 per patient and therefore will play a key role in modern cardiac surgery in the near future.

## Background

Rehabilitation process targets to counteract the disease- or intervention-induced physical disability. The primary aim of the rehabilitation process is to achieve the preexisting functional capacity and to restore the physical health. Early reestablishment of functional capability, faster convalescence and reintegration into everyday life are the equivalents of quality of life after the surgery and, therefore, the primary objectives of every single patient [1]. Simultaneous healthcare cost control is the objective of individual hospitals and national healthcare systems.

One of the major advances in the modern perioperative medicine was the establishment of Fast-Track or Enhanced Recovery After Surgery (ERAS) protocols in individual surgical disciplines [2]. An ERAS protocol is a multidisciplinary and integrative approach that combines (a) an optimization in the preoperative physical and mental health state, (b) an improvement of intraoperative management through standardized minimally invasive surgery protocols, (c) early extubation and an implementation of optimized postoperative recovery protocol integrating an individualized analgesia and a proactive physiotherapy program. Increasing implementation of such protocols has led to the origination of an international ERAS society and the dissemination of ERAS protocols in various surgical disciplines [3]. Following the first established protocols in colorectal surgery [4], the ERAS Society developed and certified additional protocols in the pancreas surgery [5], as well as in urology [6] and gynecology [7].

Although ERAS protocols have been largely established in most surgical disciplines, cardiac surgery remained unaffected in this regard, with only few modified aspects with reference to fast-track surgery [8–10]. A recently published meta-analysis showed that low-dose opioid-based anesthesia and an early extubation do not increase perioperative complication rate and significantly reduce the length of the intensive care unit (ICU) stay. In 2019, ERAS guidelines for cardiac surgery have been published [11] that highlight the importance of standardized ERAS protocol implementation in cardiac surgery to find the right balance between patients’ safety and economic resources. We aimed to analyze the economic impact of the newly established ERAS protocol in minimally invasive heart valve surgery at our institution.

## Methods

### Patients

After approval by our local Ethics Committee (PV7050), all consecutive patients who underwent an elective minimally-invasive heart valve surgery (i.e., mitral or aortic valve repair/replacement) and agreed to participate were included in our newly developed ERAS protocol.

In this pilot study, 130 consecutive patients were included for primary analysis. Out of those 130 patients, 61 patients were treated with the ERAS protocol between February 1st, 2018 and March 31st, 2019 and were implemented in the analysis (**ERAS-group**). During the same time period a total of 69 patients underwent elective minimally-invasive heart valve surgery who did not participate in the ERAS-program. Those patients were managed according to the hospital standards and served as a control group (**Control-group**). Exclusion criteria for participation in the ERAS program as well as for the Control-group were an age > 70 years, concomitant procedures requiring complete sternotomy access as well as the lack of ability (i.e., frailty) or willingness to participate at the high-intensity physiotherapy protocol.

### ERAS-protocol

The ERAS protocol was developed in accordance to the ERAS guidelines for cardiac surgery [11] and in collaboration of an interdisciplinary and interprofessional group of experts at our University Medical Center. This group of experts consisted of cardiac surgeons, cardiovascular anesthesiologists, cardiologists, perfusionists and physiotherapy specialists. Those experts defined an integrative perioperative ERAS protocol which complies with the current guidelines for perioperative cardiovascular medicine and consists of 3 major parts:

1. Preoperative optimization

The ERAS program starts with an interdisciplinary patients’ consultation 2–3 weeks before the scheduled operation by a team including cardiac surgeon, physiotherapist, nursing staff, psychologist and the rehabilitation team. All patients undergo a formal assessment of their current physical condition (frailty assessment using the LUCAS scale [12] and assessment of functional capacity with the 6-min walk test [13]) and their suitability/motivation to participate in the ERAS program. In addition, the patients are introduced into the specific points of the ERAS program, with a particular focus on physiotherapeutic exercising. Patients are asked to intensify daily training activities two to 3 weeks before the surgery and mprove their nutritional status if required. In the last 2 weeks before surgery, nutritional support through an energy-and carbohydrate-rich diet is recommended. The patients are then admitted 1 day prior to surgery in order to complete preoperative diagnostics.

2) Intraoperative management

The surgery is performed using a minimally invasive access to reduce the surgical trauma. Aortic valve surgery is performed via a partial upper sternotomy while mitral valve surgery is done through a right anterolateral mini-thoracotomy using a 3D endoscope (*Aesculap* 3D EinsteinVision® System). In addition, cardiopulmonary bypass flow is held constantly at 3.2 l m^2^/min and restrictive fluid substitution is administered to avoid postoperative edema. Intraoperatively, the anesthesiologic management includes for example extended cerebral monitoring, fast metabolizing anesthetic agents, additional regional analgesia and antiemetic prophylaxis. In addition, low dose antiarrhythmic prophylaxis with amiodarone is administered to prevent postoperative arrhythmias.

3) Postoperative protocol

All patients are extubated in the operating room before transfer to the recovery room. In the recovery room, intermittent non-invasive mechanical ventilation is routinely used during the first postoperative hours. Pain therapy is also carried out according to a standardized protocol. In addition, regional anesthesia with a local anesthetic is continued until the first postoperative morning in patients after minimally invasive mitral valve surgery. All patients receive their first postoperative physiotherapy treatment in the recovery room 2–3 h after the operation which includes breathing exercises and active mobilization in the sitting and upright position. All patients in this pilot project were brought to the intensive care unit (ICU) overnight after their stay in the recovery room for safety and surveillance reasons. In the next step of our program, an overnight stay in the Post Anesthesia Care Unit (PACU) unit is anticipated. In the ICU, patients receive their second physiotherapy session in the evening guided by ICU nursing staff. If justifiable, all drains and venous/arterial catheters are removed in the first postoperative morning, approximately 12 h after surgery. If uneventful, all patients are transferred to the postoperative ward in the first postoperative morning. Physiotherapeutic exercises are extended until the 3rd to 4th postoperative day while postoperative pain medication is maintained according to the standardized ERAS protocol. Due to the intensified physiotherapy, ERAS patients are discharged from the hospital on the fourth to fifth postoperative day.

### Perioperative management of control-group

The control-group is treated according to hospital standards. The patient is admitted 1 day prior to surgery in order to complete preoperative diagnostics. As well as in the ERAS-group, surgery is performed via a minimally invasive access. After surgery, all patients are transferred to the ICU with extubation a couple of hours after admission. First postoperative physiotherapy treatment is performed in the morning of the first postoperative day. In case of no events, all patients are transferred to the postoperative ward on the first postoperative day and are discharged from the hospital on the fifth to seventh postoperative day.

### Cost-analysis

For this pilot study a retrospective detailed cost comparison analysis from a hospital perspective was carried out using a micro-costing approach. This approach enables detailed performance evaluation and measurement and is very accurate in determining in-hospital costs. As part of this analysis, in-hospital days have been split up into distinct components including surgery, used materials, medication, diagnostics, and postoperative care. As opposed to that, the macro-costing approach analyzes only goods and services (such as in-hospital days) without any further differentiation. The primary study outcome was the difference in the average costs per treatment case in the ERAS vs Control group. In order to determine the costs of a treatment case, the resource consumption is determined based on the clinic documentation. This specifically included material costs (e.g. heart valve prosthesis) which were then valued monetarily using the market prices in October 1st 2019 (e.g. purchase prices,). The personnel costs (e.g., operating theater staff, other medical and nursing staff) and length of stay in the ICU and the normal ward were calculated according to the internal hospital budgeting. The fixed costs are calculated by minute (e.g. surgeons, operating room) or by hour (intensive care unit). The collected quantities In addition, the costs for internal activity allocation (IAA) were considered. This is a form of secondary cost allocation and the consequence of an internal hospital budgeting which is an essential part of operational controlling. With the help of the IAA, it is possible to determine the secondary costs in relation to the patient, in order to be able to estimate the relationship between expenditure and revenue and thus to comply with the principles of German-diagnosis related groups (G-DRG). German healthcare system follows the G-DRG system for hospital financing and for the treatment of its patients. This system was implemented in 2004 and enables in-patient hospital services to be billed according to the consistent, performance-oriented and flat-rate compensation G-DRG system. This results in a full reimbursement of the hospital for most procedures. If a procedure exceeds the G-DRG gerenerated compensation, the hospital has to pay the difference. To estimate the proceeds of the micro-costing analysis, in addition to the costs described above, the compensation received via the G-DRG system was also analyzed per patient.

### Statistical analysis

Standard definitions were used for patient variables and outcomes. Categorical variables are expressed as frequencies. Percentages were compared using the chi-square test or Fisher’s exact test, as appropriate. Continuous variables are presented as mean ± standard deviation and were compared using Student’s t-test or the Mann–Whitney test, as appropriate. All reported *p*-values are two-sided and p-values < 0.05 were considered statistically significant. All statistical analyses were accomplished with the IBM SPSS 23 software (IBM Corp., New York, USA).

## Results

### Patient characteristics

Overall, both study groups included a relatively young patient cohort without significant differences in mean age (ERAS-group: 50.7 ± 12.9 years vs Control-group: 55.5 ± 9.9 years; *p* = 0.096). All remaining demographical characteristics and cardiovascular risk factors (e.g., diabetes mellitus, dyslipidemia) were comparable in both study groups (Table 1). The most common comorbidity in both study groups was arterial hypertension (*p* = 0.94). Both studied patient groups had a low and comparable perioperative risk score according to the European system for cardiac operative risk evaluation 2 (EuroSCORE) (ERAS-group: 0.836% vs Control-group: 0.774%; *p* = 0.42). All ERAS patients were prepared for the surgery according to the preoperative ERAS protocol.

**Table 1:** Perioperative data

Variables	ERAS-group (***n*** = 61)	Control-group (***n*** = 69)	***p*** - value
Mean age (years)	50.7 ± 12.9	54.1 ± 9.5	0.096
Male sex	47 (77%)	52 (75%)	0.813
Arterial hypertension	31 (50.8%)	35 (51%)	0.936
Hyperlipidemia	18 (30%)	11 (16%)	0.055
Diabetes mellitus	1 (2%)	7 (9%)	0.156
EuroSCORE II (in %)	0.837	0.774	0.416
Duration of surgery	220.7 ± 45.6	239.6 ± 59.8	**0.047**
Mitral valve surgery	24 (39%)	34 (49%)	0.256
Aortic valve surgery	37 (61%)	35 (51%)	0.256
Intensive care unit stay (d)	1.5 ± 1.1	2.0 ± 1.9	**0.035**
In-hospital stay (d)	6.1 ± 2.6	7.7 ± 3.8	**0.008**

Intraoperatively, all patients underwent an elective minimally invasive aortic or mitral valve surgery. In the ERAS-group, 39.3% patients had a full endoscopic mitral valve surgery through a right mini-thoracotomy compared to 49% in the Control-group (*p* = 0.26). Overall, 57 patients (98%) received a mitral valve repair and only in one case the valve was replaced by a bioprosthesis. Further, most of the patients presented with primary mitral regurgitation (52/58 patients) and 6 patients had secondary mitral regurgitation. Ejection fraction of the patients with mitral valve disease was similar in both studied groups (ERAS group: 54.0 ± 15.7% vs. Control group: 57.3 ± 9.4%; *p* = 0.337). The remaining patients underwent minimally invasive aortic valve surgery through a partial upper sternotomy. Overall, 39 patients presented with predominantly aortic valve regurgitation and a successful aortic valve repair was possible in all patients. The remaining 33 patients received an aortic valve replacement due to severe aortic valve stenosis. Ejection fraction of the patients with aortic valve disease was similar in both studied groups (ERAS group: 57.7 ± 6.9% vs. Control group: 58.6 ± 8.5%; *p* = 0.657).

### Perioperative data

There were no intraoperative complications or events related to the ERAS protocol. There were also no complications associated with the ERAS protocol during the postoperative course. One patient had redo surgery in the first 24 h for relevant bleeding after minimally invasive mitral valve repair, while another patient developed severe delirium in the recovery room, which was treated medically. Other postoperative complications (e.g., postoperative atrial fibrillation) occurred with a similar frequency in both cohorts. In-hospital mortality was 0% in both groups. According to the ERAS protocol, the patients were extubated in the operating room and transferred to the recovery room. Length of ICU stay was significantly shorter in the ERAS- group as compared to the Control-group (i.e., 26.5 ± 25.2 h vs 46.6 ± 44.9 h, respectively, *p* = 0.010). Furthermore, the length of stay in the general ward (4.7 ± 2.2 vs 5.7 ± 2.7 days; *p* = 0.023) as well as the total in-hospital stay (ICU and general ward) were significantly shorter in the ERAS-group (6.1 ± 2.6 vs 7.7 ± 3.8 days; *p* = 0.007) (Table 1).

### Cost analysis

#### Operating room (OR)

**Fig. 1:**
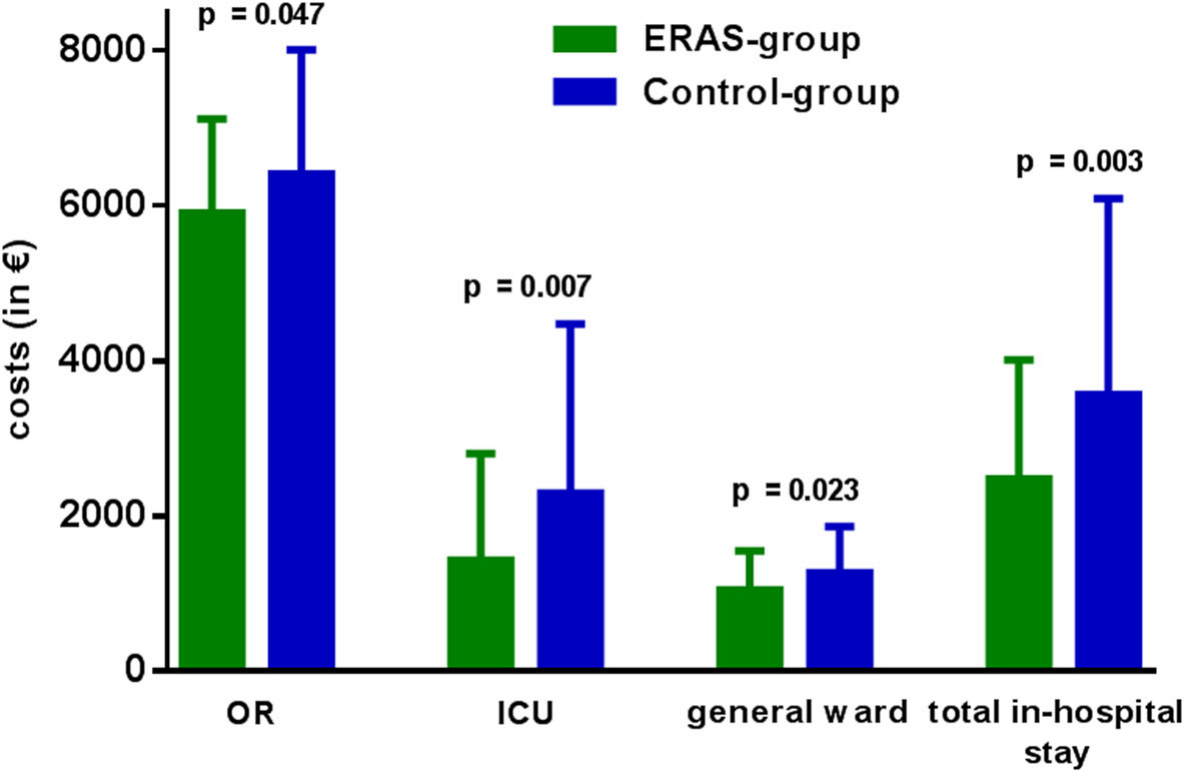
Comparison of costs per patient (in €) for the surgery (OR) intensive care unit (ICU), general ward and duration of total inpatient stay: ERAS group (*n* = 61) vs Control group (*n* = 69); (* *p* < 0.05; ** *p* < 0.005; *** *p* < 0.001)

The fixed costs for the operating room (OR) are € 25.0 per minute. Further, the fixed personnel costs during the surgery are € 1.75 (€ 1.15 per minute for the cardiac surgery team as well as € 0.60 per minute for the OR nurse team). Total OR time was significantly shorter in the ERAS-group as compared to the Control group (i.e., 220.7 ± 45.6 min vs 239.6 ± 59.8 min; *p* = 0.047). Accordingly, mean OR costs/case were € 5518.4 ± 1.140.1 in the ERAS-group which was € 472.5/case less when compared to the Control-group (€ 5990.9 ± 1495.5; *p* = 0.047). In addition, significantly shorter surgery times led to lower OR personnel costs (€ 386.2 ± 79.8/case in the ERAS-group vs € 419.4 ± € 104.7/case in the Control-group, *p* = 0.047). Overall, the total OR costs were € 5904.7 ± 1219.9/case for the ERAS-group vs € 6410.3 ± 1600.1/case in the control group (*p* = 0.047) (Fig. 1). In direct comparison, purchase costs for the implemented surgical materials were similar in both studied groups (ERAS-Cohort: € 723.5 ± 717.6 per patient vs Control-group: € 877.1 ± 525.8; *p* = 0.163).

#### Hospital stay

In the ERAS-group the time from the end of the skin suture until extubation were 21.8 ± 11.5 min and significantly shorter compared to the ventilation time of the control group (371.3 ± 129.5 min); *p* < 0.001. The ICU stay is charged with € 50/h. The significantly shorter ICU stay in the ERAS-group vs Control-group (i.e., 26.5 ± 25.2 h vs 46.6 ± 44.9 h, respectively, *p* = 0.010) resulted in a significant cost reduction by € 925.7 in the ERAS group (€ 1431.9 ± € 1369.2 in the ERAS-group vs € 2294.9 ± € 2185.5 in the Control-Group; *p* = 0.007) (Fig. 1).

A similar trend was observed in the length of stay on the general ward. The costs for cardiac surgical general ward treatment at the University Heart and Vascular Center are € 225/day. The mean length of stay on the general ward was 4.7 ± 2.2 days in the ERAS-group which resulted in costs of € 1047.5 ± 504.6/patient in the ERAS group. In the Control-group, the mean length of general ward stay was 5.6 ± 2.7 days with resultant costs of € 1271.2 ± 596.3/patient. The cost savings for the general ward stay were € 223.7 per patient in the ERAS cohort (*p* = 0.023).

In terms of total in-hospital stay (i.e., ICU and normal ward), the implementation of ERAS protocol **(**Fig. 1) resulted in a significant cost advantage of € 1087.2 per patient (€ 2479.5 ± 1537.3 vs 3566.7 ± € 2528.7; *p* = 0.003) (Fig. 1).

#### Internal activity allocation (IAA)

**Fig. 2:**
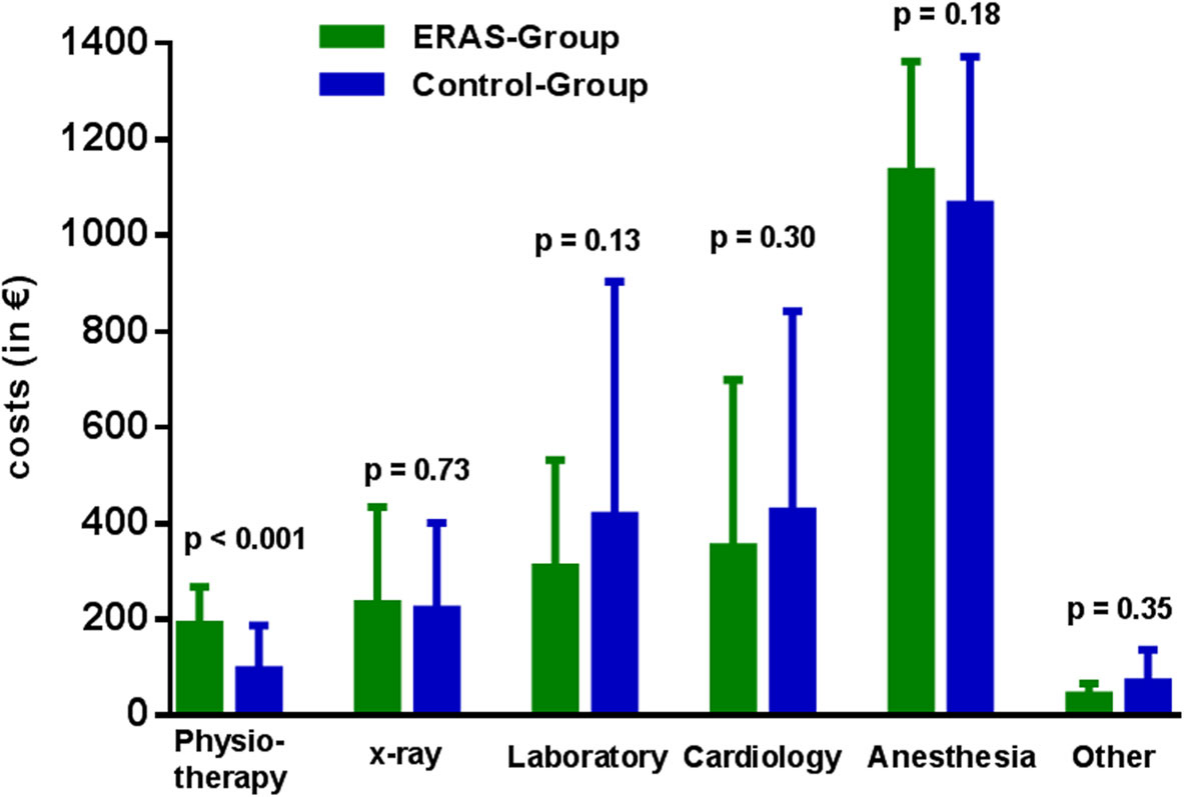
Comparison of costs per patient (in €) for internal activity allocation (IAA): ERAS group (*n* = 61) vs Control group (*n* = 69); (*p < 0.05; **p < 0.005; ***p < 0.001)

The total costs for the IAA are displayed per patient in the Fig. 2 and were comparable in both groups (ERAS-group: € 2092.3 ± € 800.3 vs Control-group: € 2255.8 ± € 995.3; *p* = 0.31). Most relevant part of IAA costs were due to anesthesia (i.e., physician and nurse) which represented more than the half of IAA costs in both study groups (*p* = 0.18). Furthermore, the costs of the echocardiography (*p* = 0.30), the laboratory tests (*p* = 0.13) and X-ray diagnostics (*p* = 0.73) were not significantly different between the two study groups. The only significant difference were higher costs due to physiotherapy costs in the ERAS-group which resulted in additional costs of € 94.3 per patient as compared to the Control-group (ERAS-group: € 188.8 ± 78.7 vs control group: € 94.5 ± 92.9; *p* < 0.001) (Fig. 2).

#### Costs

After a detailed cost analysis, the total in-hospital stay costs in the ERAS cohort were € 2479.5 ± 1537.3/patient. The purchase costs for the implemented surgical material (€ 723.5 ± 717.6/patient), the total OR costs (€ 5904.7 ± € 1219.9/patient) and the additional IAA costs (€ 2092.3 ± € 800.3/patient) resulted in total costs of € 11,200.0 ± 3.029.6 per patient in the ERAS-group.

In the Control-group, total in-hospital stay costs were € 3566.7 ± 2528.7/patient. The addition of costs for the implemented surgical material (€ 877.1 ± 525.9/patient), total OR costs (€ 6410.3 ± 1600.1/patient) and the IAA costs of € 2255.8 ± 990.3 resulted in a total cost of € 13,109.8 ± 4527.5 per patient in the Control-Group. As a final result of costs analysis, the implementation of the ERAS program in our hospital resulted in a financial advantage of € 1909.8 per patient (*p* = 0.006). The main factor of the cost savings was the reduction in the in-hospital stay with a resultant cost savings of € 1087.2 per ERAS-patient. The only cost increase in the ERAS-group is represented by additional costs of € 94.3 due to intensified physiotherapy protocol. In summary, the establishment of ERAS protocol in or hospital resulted in total costs savings of € 116,497.8 in the pilot phase of ERAS project.

## Discussion

Modern medicine balances in the conflict to find the right compromise between patient safety and available economic resources. ERAS concept is becoming an increasingly important milestone in the modern perioperative medicine and has already been adopted by many surgical disciplines [4, 5, 14–16]. Historically, almost 20 years ago, first attempts were made to implement so-called fast-track protocols in the modern cardiac surgery. The initial attempts of fast-track protocols were already evident in the 1990s by introducing early extubation protocols to improve the recovery after cardiac surgery [17]. In 1996, Falk et al. proposed that low-risk patients could be identified with the help of a scoring system and could be early transferred from ICU to low-dependency postoperative care units. According to the authors, this fast-track postoperative care could lead to significant cost savings [18]. In addition, London et al. described in 1997 a fast-track protocol, which provided an early extubation and a shortening of the intensive care unit stay in cardiac surgery. The implementation of such protocol led to a significant reduction in the length of hospital stay without simultaneously increasing the 30- and 6-month mortality. Moreover, early extubation led to a significant reduction in nosocomial infections [19]. Further, Chen et al. described in 1998 the concept of “fast-track Anesthesia”, which aimed for extubation within 1–6 h after surgery, as well as the concept of “fast-track Heart Surgery” which was a multidisciplinary approach to improve the efficiency in the treatment of cardiac surgery patients [20]. These historical protocols were primarily focused on the intraoperative management and showed the potential for early extubation in the intensive care unit, a shorter ICU stay and a potential for costs reduction in cardiac surgery.

Despite these promising initial attempts and the further development of minimally invasive cardiac surgery as well as introduction of numerous catheter-based cardiovascular interventions (e.g. transcatheter aortic valve replacement), the ERAS protocols in cardiovascular medicine are still in their infancy. The first recommendations for ERAS implementation in cardiac surgery were published by Engelman et al. in August 2019 [11]. Standardized perioperative processes through the ERAS protocol in cardiac surgery can be expected to improve patient safety and quality of care with additional cost savings. Recently published randomized study from China [21] and an observational study from the USA [22] compared the ERAS concept with the conventional standard of care in cardiac surgery. In accordance with the previous studies in other surgical disciplines, both above-mentioned studies showed a significantly improved perioperative outcome with less perioperative complications and shorter ICU- and in-hospital stay after cardiac surgical procedures. Quite similar, we had no ERAS-associated complications in our pilot project at our institution. Furthermore, we aimed to examine the economic impact of ERAS program implementation at an university medical facility by performing a detailed cost analysis in comparison with a conventional treatment group. We could convincingly demonstrate a significant cost saving effect in the ERAS program in cardiac surgery. Costs saving resulted predominantly from the cost reduction in the ICU- and in-hospital stay with a final cost savings of € 1909.8 per ERAS-patient. Furthermore, the post-hoc analysis showed that the implementation of the ERAS program at our institution resulted in the mean reduction of in-hospital stay by 1.57 days per ERAS-patient. The reduction of in-hospital days in the ERAS-group was seen although 10% fewer ERAS-patients were treated via a right mini-thoracotomy compared to the control-group. A procedure without a sternotomy usually results in a shorter in-hospital stay [23]. This fact highlights even more the benefit of the ERAS program: Although more ERAS patients have been treated with a partial-upper sternotomy, the ERAS-patients are discharged earlier from the hospital compared to the control group resulting in a total saving of 95.77 patient-bed days in the initial 61 ERAS patients. Considering that the mean length of stay of ERAS patients was 6.11 days, 15.67 additional ERAS patients could be potentially treated for those 61 ERAS-patients.

In summary, our pilot study showed that implementation of ERAS protocol in the initial 61 patients resulted in total costs savings of € 116,497.8. In the long run, those cost savings may result in further investments in other parts of the health system and therefore increasing the economic benefit of the ERAS program. Overall, we could convincingly demonstrate that implementation of ERAS protocol has a clear potential to improve patient care in cardiac surgery while saving costs. Due to new cost-intensive technologies, increasing patient expectations and demographic changes in the population, there is an urgent need for improvements in the healthcare system. According to our study, the implementation of ERAS concept could be one of such key strategies in the near future. Modern cardiac surgery should consider integrating ERAS-protocols into the daily clinical practice to find the right balance between patient safety and available economic resources and to comply with the recent advances in the modern cardiovascular medicine. The crucial component and the key of success in such ERAS protocols is a multi-professional and interdisciplinary collaboration in the integrated patient care. Therefore, the establishment of an interdisciplinary ERAS team is essential to ensure an interprofessional exchange for continuous process optimization on a regular base. The ERAS team should be a crucial interface between the various disciplines helping to define institutional protocols and internal guidelines for perioperative medicine.

### Limitations

This is a retrospective analysis of first 61 patients that entered our ERAS program compared to a cohort of standard care with all known limitations of such a study design. Furthermore, the present study has been a single-center experience and therefore does not allow any generalization. In addition, since the studied patients were not randomized, we cannot exclude, that some of the differences are due to the selection bias. However, since both studied groups were treated via a minimally-invasive access and were comparable in regards of age and preoperative risk factors (e.g. EuroSCORE II; see also Table 1) we believe that the differences are mainly driven by the positive effect of the ERAS-program resulting in enhanced postoperative recovery. Further, we represent our pilot project with ongoing modifications during the initial implementation and the effect of “learning curve”. Therefore, this pilot study included only younger patients without out major comorbidities which were treated via a minimally-invasive access. This was necessary to implement the new ERAS protocol in our hospital institution in order to assure that no severe adverse events occur.

Furthermore, we should consider the fact that by reducing the length of hospital stay, we may transfer some costs to other health care sectors. These subsequent costs, including the long-term cost effects of ERAS program implementation, were not analyzed in the present study, and will be addressed by an ongoing randomized controlled study at our institution. Furthermore, the impact of ERAS program to the postoperative quality of life was not assessed in our study. However, this will also be evaluated by the ongoing randomized controlled study.

## Conclusion

Enhanced Recovery After Surgery (ERAS) is an evidence-based method for optimizing perioperative processes in the context of various surgical interventions. This study shows that the implementation of an ERAS-protocol in minimally-invasive cardiac surgery can be carried out safely with a fast postoperative recovery of the patient. ERAS results in a cost savings of up to € 1909 per patient and therefore will play a key role in modern cardiac surgery in the near future.

## Data Availability

The datasets used and analysed during the current study are available from the corresponding author on reasonable request.

## Abbreviations

ERAS: Enhanced Recovery After Surgery
G-DRG: German-diagnosis related groups
IAA: Internal activity allocation
ICU: Intensive care unit
